# Precise CRISPR-Cas9 Mediated Genome Editing in Super Basmati Rice for Resistance Against Bacterial Blight by Targeting the Major Susceptibility Gene

**DOI:** 10.3389/fpls.2020.00575

**Published:** 2020-06-12

**Authors:** Kashaf Zafar, Muhammad Zuhaib Khan, Imran Amin, Zahid Mukhtar, Sumera Yasmin, Muhammad Arif, Khansa Ejaz, Shahid Mansoor

**Affiliations:** ^1^Agricultural Biotechnology Division, National Institute for Biotechnology and Genetic Engineering, Constituent College of Pakistan Institute of Engineering and Applied Sciences, Faisalabad, Pakistan; ^2^Department of Biotechnology, Balochistan University of Information Technology, Engineering and Management Sciences, Quetta, Pakistan; ^3^Soil and Environmental Biotechnology Division, National Institute for Biotechnology and Genetic Engineering, Constituent College of Pakistan Institute of Engineering and Applied Sciences, Faisalabad, Pakistan

**Keywords:** genome editing, CRISPR-Cas9, rice improvement, bacterial blight, *Xanthomonas oryzae*

## Abstract

Basmati rice is famous around the world for its flavor, aroma, and long grain. Its demand is increasing worldwide, especially in Asia. However, its production is threatened by various problems faced in the fields, resulting in major crop losses. One of the major problems is bacterial blight caused by *Xanthomonas oryzae pv. oryzae (Xoo)*. *Xoo* hijacks the host machinery by activating the susceptibility genes (*OsSWEET* family genes), using its endogenous transcription activator like effectors (TALEs). TALEs have effector binding elements (EBEs) in the promoter region of the *OsSWEET* genes. Out of six well-known TALEs found to have EBEs in Clade III *SWEET* genes, four are present in *OsSWEET14* gene’s promoter region. Thus, targeting the promoter of *OsSWEET14* is very important for creating broad-spectrum resistance. To engineer resistance against bacterial blight, we established CRISPR-Cas9 mediated genome editing in Super Basmati rice by targeting 4 EBEs present in the promoter of *OsSWEET14*. We were able to obtain four different Super Basmati lines (SB-E1, SB-E2, SB-E3, and SB-E4) having edited EBEs of three TALEs (*AvrXa7*, *PthXo3*, and *TalF*). The edited lines were then evaluated in triplicate for resistance against bacterial blight by choosing one of the locally isolated virulent *Xoo* strains with *AvrXa7* and infecting Super Basmati. The lines with deletions in EBE of *AvrXa7* showed resistance against the *Xoo* strain. Thus, it was confirmed that edited EBEs provide resistance against their respective TALEs present in *Xoo* strains. In this study up to 9% editing efficiency was obtained. Our findings showed that CRISPR-Cas9 can be harnessed to generate resistance against bacterial blight in indigenous varieties, against locally prevalent *Xoo* strains.

## Introduction

In plants, different genome editing strategies have been exploited including zinc finger nucleases, transcription activator-like effector nucleases (TALEs), and clustered regularly interspaced short palindromic repeats (CRISPR) and CRISPR associated (Cas) nucleases. Among these tools, the RNA guided CRISPR-Cas9 system has become the method of choice for genome editing because of its simplicity, ease of performing, and versatility. This system exploits the complementary base pairing mechanism of DNA to guide site-specific Cas9 endonuclease to the target site. The guide RNA (gRNA) screens the template, recognizes the specific complementary target sequence, and signals to Cas9 to introduce a double stranded break (DSB) at the target site. A triplet of nucleotides (NGG) at the 3′ end of the target site, also known as protospacer-associated motif (PAM), is essential for Cas9 to introduce a double stranded break (DSB) 3 bp upstream of the PAM sequence. These DSBs are repaired either via imprecise non-homologous end joining (NHEJ) or template directed precise homology directed repair (HDR) (Barrangou et al., 2007; Cong et al., 2013; Mali et al., 2013; Hsu et al., 2014; Wright et al., 2016). Until today, this technology has been successfully used to engineer resistance against various pathogens and for agronomic trait enhancement.

**FIGURE 1:**
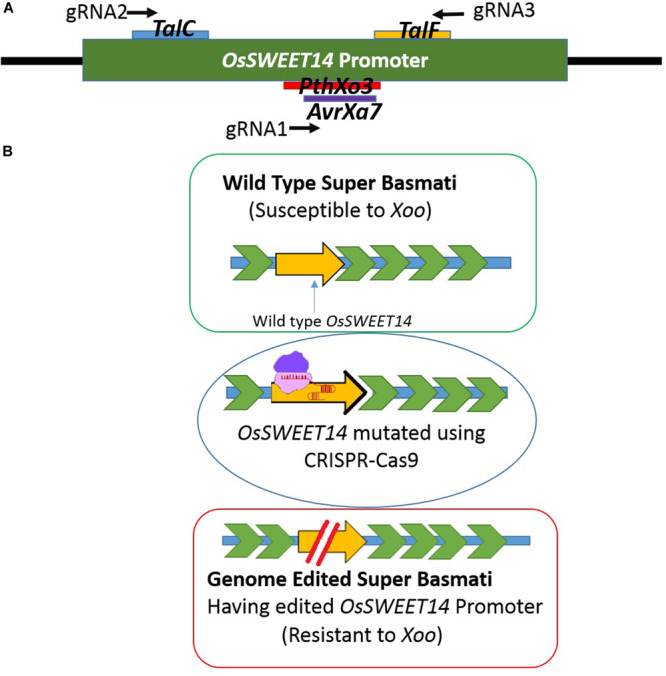
**(A)** Schematic figure of the promoter showing EBEs location on the promoter against which three gRNAs were designed. gRNA1 was designed to target *PthXo3* and *AvrXa7*, gRNA2 for *TalC* and gRNA3 targeted *TalF.* **(B)** Schematic figure of developing resistance by editing the *OsSWEET14* gene. The intact promoter was susceptible to *Xoo* whereas the EBE edited promoter can be resistant to *Xoo*.

Rice is a staple food crop which belongs to the family Poaceae, genus *Oryza*. It has been cultivated for more than 10,000 years and is the second most commonly cultivated cereal in the world (Sasaki, 2001; Pazuki and Sohani, 2013). For Asian countries, it contributes 50–80% of daily calories and serves more than 90% of the population (Khush, 2005; Zeigler and Barclay, 2008). Among different varieties of rice, Basmati rice is famous around the globe for its flavor, aroma, and long grain. Due to food insecurity, rice demand is increasing every day (Shobarani et al., 2010). However, Basmati rice production is threatened by various problems in the field resulting in major crop losses. One of the grave problems is bacterial blight caused by *Xanthomonas oryzae pv. Oryzae (Xoo)*. It is the most destructive and deadly bacterial disease and can cause up to 75% crop loss. *Xoo* hijacks the host machinery by activating the susceptibility genes (*SWEET* family genes), using its endogenous transcription activator like effectors (TALE). TALEs have their effector binding elements (EBEs) in the promoter region of *OsSWEET* genes. These effectors divert sugars from the plant cell to fulfill the pathogen’s nutritional needs (Chen et al., 2012). Most of the geographically distinct *Xoo* strains target *OsSWEET14*, which encodes the sucrose-efflux transporter family (Chen et al., 2010). There are six known TALEs which target promoter regions of *OsSWEET* genes, and the EBEs for four different TALEs, *PthXo3, AvrXa7*, *TalC*, and *TalF* (previously known as Tal5) are present in *OsSWEET14* (Antony et al., 2010; Yu et al., 2011; Streubel et al., 2013; Oliva et al., 2019) (Figure 1A).

Rice has been used extensively as a model for performing genome editing studies via CRISPR-Cas9 due to its diploid nature (Jiang et al., 2013; Hu et al., 2016; Abe et al., 2018). There are some prior studies where genome editing has been done to develop resistance against bacterial blight using different genome editing platforms including CRISPR-Cas9 (Blanvillain-Baufumé et al., 2017; Oliva et al., 2019; Xu et al., 2019). Customization of the CRISPR-Cas9 tool offers an avenue to target and mutate TALE binding elements in the promoter region of *OsSWEET14* to resist the spread of bacterial blight (Jiang et al., 2013). Different types of natural mutations have been reported in *OsSWEET14* genes in different rice cultivars which provide immunity against *Xoo* strains (Hutin et al., 2015b). However, no natural mutations have been reported in Super Basmati rice (Zaka et al., 2018). Here, we have employed CRISPR-Cas9 technology to engineer resistance against bacterial blight in Super Basmati rice for the first time by targeting the promoter region of the *OsSWEET14* gene (Figure 1B).

## Materials and Methods

### gRNAs Design and Construct Development

**FIGURE 2:**
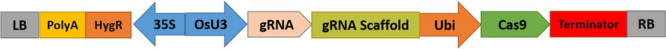
Schematic diagram of the construct used for genome editing. All three gRNAs were cloned in the same way. The construct was expressing gRNA under the *OsU3* promoter. The rice codon-optimized Cas9 was expressing under the *Ubi* promoter.

The genome sequence of Super Basmati rice was not available in nucleotide databases. To know the exact sequence of the *OsSWEET14* gene promoter and to design gRNAs, different primer sets (*Os*P-F: 5′ ATTGGCACTTTCTGTCATGCATG 3′ and *Os*P-R: 5′ GCAAGATCTTGATTAACTAGCTAGC 3′) were first designed on the *OsSWEET14* gene promoter based on the sequences of other rice varieties available in the database. The genomic DNA of Super Basmati rice was extracted using the cetyl trimethylammonium bromide (CTAB) method (Stewart, 1993) followed by polymerase chain reaction (PCR). An amplicon of 457 bp in length was cloned into a pTZ57 R/T (Thermo Fisher Scientific, United States) vector and sent for sequencing. All gRNAs were designed manually to target their respective EBEs (Table 1). The overhangs of GGCA and AAAC were added a 5′ end of forward and reverse gRNA respectively for cloning at the *Bsa*I site (Table 2). The gRNAs were screened for potential off-targets using the online tool Cas-OFFinder^1^. Analysis revealed that there were no off-targets. The gRNAs were synthesized, annealed, and cloned at the *Bsa*I site in pRGEB32 (Addgene plasmid # 63142) under the rice *U3* promoter (Xie et al., 2015). The pRGEB32 already has rice codon optimized Cas9 expressed under the *Ubi* promoter. The constructs with the Cas9 gene and gRNA cassette (shown in Figure 2) were confirmed by PCR and restriction followed by Sanger sequencing. The Cas9 gene was confirmed by PCR using specific primers (Cas9-F 5′ AGCATCGGCCTGGACATCGGC 3′ and Cas9 R- 5′ CCGGAACTTGATCATGTGGG 3′). The full length Cas9 was also confirmed in constructs using primer set 3-full Ca9 (Supplementary Table S1).

**TABLE 1:** Sequences of EBEs present in the *OsSWEET14* promoter region.

TALE EBE	Sequence	References
***AvrXa7***	ATAAACCCCCTCCAACCAGGTGCTAA	Antony et al., 2010
***PthXo3***	ATATAAACCCCCTCCAACCAGGTGCTAAG	Antony et al., 2010
***TalF***	TAAGCTCATCAAGCCTTCA	Streubel et al., 2013
***TalC***	CATGCATGTCAGCAGCTGGTCAT	Yu et al., 2011

**TABLE 2:** Sequences of gRNAs to target EBEs of the *OsSWEET14* promoter region.

gRNA	Sequence	Target
*OsP*gRNA1	F: 5′-GGCA G ATATAAACCCCCTCCAACC-3′ R: 5′-AAAC GGTTGGAGGGGGTTTATAT C-3′	*AvrXa7, PthXo3*
*OsP*gRNA2	F: 5′-GGCA GGGCATGCATGTCAGCAGC-3′ R: 5′-AAAC GCTGCTGACATGCATGCCC-3′	*TalC*
*OsP*gRNA3	F: 5′-GGCA G TGAGTTTGCTTTGCTTGAA-3′ R: 5′-AAAC TTCAAGCAAAGCAAACTCA C-3′	*TalF* (Previously *Tal5*)

*Red bases show the overhangs added to make gRNAs compatible for cloning in pRGEB32.*

### Plant Material

Super Basmati (Pakistan’s indigenous rice variety) was used to establish genome editing against bacterial blight. The rice variety IR24 was used as a susceptible control. Rice seeds were obtained from DNA Markers and the Applied Genomics Lab of National Institute for Biotechnology and Genetic Engineering (NIBGE), Faisalabad, Pakistan. The seeds were manually de-husked, and surface sterilized with 70% ethanol followed by dipping in 50% (v/v) commercial bleach (having 5.25% sodium hypochlorite). After washing with distilled water, seeds were placed on MS media (Murashige and Skoog, 1962) supplemented with vitamins and 2,4-Dichlorophenoxyacetic acid (2,4-D) for callus formation.

### Rice Transformation and Growth Conditions

The 28 days old embryogenic calli were selected and placed on an osmotic medium with 0.25 M mannitol. The constructs with gRNA and Cas9 were confirmed again and coated on 1 μm gold particles (Bio-Rad, United States) for biolistic transformation. The rice calli were transformed through bombardment by a gene gun (PDS-1000/Bio-Rad, United States) following optimized protocol for biolistic transformation of Super Basmati rice (Mukhtar and Hasnain, 2018). For each gRNA approximately 4,000 calli were transformed. The transformed calli were placed at 28–30°C for 24 h in a dark room under sterile conditions. After 24 h, the transformed calli were shifted to the selection medium with 50 mg/L hygromycin. After 15 days they were again shifted to fresh selection medium. After completing 30 days on the selection medium, the surviving calli were shifted to pre-regeneration and then to regeneration media. The regenerated plantlets were shifted to rooting media. After the development of roots, they were transferred to soil and kept in the greenhouse under controlled conditions. After 15 days the plants were shifted to large pots and allowed to grow to maturity and the seeds were then harvested.

### Confirmation of Transgenic Rice

To confirm the presence of Cas9 and gRNA cassette, DNA was extracted from all plants that were developed through tissue culture, using the CTAB method (Stewart, 1993). The construct was confirmed by PCR using forward primer at vector backbone (OsPRGEB32 F 5′ GGTGCTACCAGCAAATGC TGGAAGCCG3′) and reverse primer designed on gRNA (*OsP*gRNA1-R: 5′-AAACGGTTGGAGGGGGTTTATATC-3′, *OsP*gRNA2-R: 5′-AAAC GCTGCTGACATGCATGCCC-3′ and *OsP*gRNA3-R: 5′-AAAC TTCAAGCAAAGCAAACTCAC-3′ for gRNA 1, 2, and 3 constructs, respectively). The 273 bp amplicon confirmed the presence of construct (Supplementary Figure S2). Gene specific primers were used to confirm the presence of Cas9 (Table 1- primer set 2 (partial Cas9) and 3 (full Cas9). Promoter fragments of the *OsSWEET14* gene were amplified using primers (Supplementary Table S1- primer set 1) from all the plants and PCR products were sent for Sanger sequencing.

### T7 Endonuclease Assay

For the T7 endonuclease assay, the genomic DNA was extracted from T_0_ plants and the flanking target region was amplified using primer set 1 (Supplementary Table S1- *Os*P-F and *Os*P-R) to amplify the promoter (Supplementary Table S1). The purified PCR amplicons were denatured and renatured then subjected to T7 endonuclease I (NEB, M0302) following the manufacturer’s instructions. The products were resolved on 2% agarose gel and then stained with ethidium bromide.

### Screening Against Bacterial Blight Resistance

The screening of edited lines against bacterial blight was performed under containment glasshouse conditions. The 1st round of screening was performed on T_0_ plants. Two plants from each line were selected for inoculation. T_1_ seeds were then collected from primary transformants. Seeds from each line were sown in separate pots containing soil (Excluding SB-E1 because seed filling was disturbed for this line). When the plants reached the three leaf stage, they were arranged in different batches for screening against bacterial blight. The experiment was conducted in three batches. There were six pots in each batch. Each pot had three plants of wild type Super Basmati, SB-E2, SB-E3, SB-E4, IR-4 (susceptible check), and a negative control. So, the edited plants were arranged along with their negative, susceptible, and wild type controls. There were three pots for each line, so a total of nine plants were inoculated for each line. The wild type Super Basmati has no editing, and the IR-24 line is susceptible to almost all kinds of *Xoo* strains, and was therefore used as a susceptible control. The negative control is the plant for which scissors were dipped in distilled water, without any *Xoo* strain, to check the lesion length introduced due to scissor injury. All of these lines were screened for bacterial blight resistance using a local virulent *Xoo* strain. This strain was selected on the basis that one of the four EBEs of *OsSWEET14* is *AvrXa7* which was present in the strain. The inoculum was prepared as described by Tu et al. (2000). All the lines were maintained in a uniform environment in the glasshouse at 30°C and 85% relative humidity. For leaf clip inoculation, 45 days old plants were used. For each plant three to four leaves were uniformly inoculated with scissors dipped in bacterial suspension (Kauffman, 1973). At 14 days post-inoculation (dpi) the plant’s responses were observed and data was recorded by measuring the full length of the leaf and lesion length in centimeters (cm). The percentage disease leaf area (%DLA) was then calculated using the below formula.

% DLA (cm)=[Lesion Length (cm)/⁢Full leaf length (cm)]∗100

### RNA Extraction, cDNA Synthesis and RT-qPCR

The 30 days old rice seedlings were inoculated with scissors dipped in *Xoo* suspension. The total RNA was extracted after 24 h post-inoculation (hpi) from rice leaves using TRIzol reagent following the manufacturer’s instructions (Invitrogen, United States). The isolated RNA was treated with DNaseI as per the manufacturer’s instructions (Thermo Fisher Scientific, United States). The complementary DNA (cDNA) was synthesized from RNA using a RevertAid first strand cDNA synthesis kit (Thermo Fisher Scientific, United States) following instructions given by the manufacturer. The induction of *OsSWEET14* by *Xoo* was determined in wild type as well as in edited plants by RT-qPCR. The *OsSWEET14* transcripts were amplified using *OsSWEET14*-specific primers OsSWEET14-RT-F/OsSWEET14-RT-*R* (Supplementary Table S1). A volume of 25 μL reaction mixture was made using 12.5 μL SYBR Green Real-Time PCR Master Mix (Thermo Fisher Scientific, United States), 0.1 pmole of forward and reverse primers, 2.5 μL cDNA (∼25 ng), and 9.5 μL water. The conditions were optimized and finally the reaction was performed using a Bio-Rad iQ5 thermal cycler (Bio-Rad, United States). The expression of sucrose phosphate synthase (SPS) was used as an internal control and was amplified using primers SPS-F/SPS-R (Supplementary Table S1). The quantification results were analyzed using the 2^–ΔΔΔCt^ method (Livak and Schmittgen, 2001). Each reaction was performed in triplicate.

### Phenotyping

The plants were observed visually during growth. After harvesting the seeds from edited plants, the germination of the seeds was checked. The seeds were dehusked, sterilized and placed in six-well plates in water and the rate of germination was recorded. This was done for all the lines except SB-E1 because we were unable to obtain seeds from this line. The root and shoot lengths were measured by growing three to four seeds from each line vertically on square plates with ½ MS media (no sucrose was added). After 1 week, the root and shoot lengths were again measured and compared with the wild type.

## Results

### Development of Constructs

The sequence of the *OsSWEET14* gene promoter was amplified using the primer set *Os*P-F/*Os*P-R (Supplementary Table S1). This primer set was designed on the sequences of other rice varieties available in the database. Upon amplification a PCR, a product of 457 bp, was amplified which was cloned and sequenced. The sequenced region of the *OsSWEET14* promoter from Super Basmati rice was submitted to Genbank and is available online under Accession No. MK791135.1. Three different gRNAs (Table 2) were designed to target EBEs (Table 1) of *AvrXa7*, *PthXo3*, *TalF*, and *TalC* in the promoter region. Due to overlapping EBEs for *AvrXa7* and *PthXo3*, the first gRNA (gRNA1) was designed to target both EBEs simultaneously. Similarly, gRNA2 and gRNA3 were designed to target *TalC* and *TalF*, EBEs, respectively.

The constructs containing the Cas9 gene and gRNA cassette were initially confirmed by PCR. Gene specific primers were used to confirm the Cas9 gene with an amplicon length of 490 bp (Supplementary Figure S3A). In the final constructs, full-length Cas9 (4100 bp) was confirmed (Supplementary Figure S3B). The gRNA cassette was confirmed using the forward primer (OsPRGEB32 F GGTGCTACCAGCAAATGCTGGAAGCCG) and reverse primer of the respective gRNA (Table 2). The 273 bp product confirmed the presence of gRNA along with its scaffold in the pRGEB32 vector (Supplementary Figure S1A). The presence of gRNAs in constructs was also confirmed by restriction analysis. gRNA insertion in pRGEB32 results in disruption of the *Bsa*I site. Due to this disruption, restriction with *Bsa*I and *Hind*III enzymes did not release 400 bp fragments in positive clones. Thus, the absence of 400 bp confirmed that all the gRNAs were successfully cloned (Supplementary Figures S1B,C). Finally, all constructs were confirmed by Sanger sequencing (Supplementary Figure S1D).

### Transgenic Plants Development

The process of transgenic plant development and screening against bacterial blight is shown in Supplementary Figures S4B,C. Cas9 expressing transgenic rice lines were developed under the *Ubi* promoter. All the three gRNAs (designated as gRNA1, gRNA2, and gRNA3) were expressed under rice U3 promoter in a pRGEB32 vector with rice codon-optimized Cas9. A total of 48 transgenic lines were obtained for gRNA1, 35 lines for gRNA2, and 51 lines for gRNA3. Both edited and wild type lines were maintained at 30°C in a containment glasshouse.

### Mutation Detection

**FIGURE 3:**
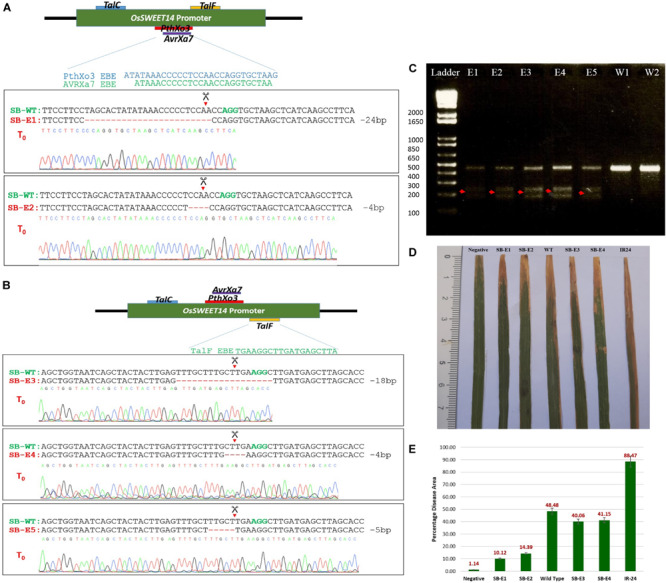
**(A)** Sanger sequencing of *AvrXa7* and *PthXo3* EBE edited plants. The plants were named as SB-WT (Super Basmati wild type), SB-E1 (Super Basmati edited 1) and SB-E2 (Super Basmati edited 2). Green colored bases AGG represents the PAM sequence and red dashes symbolize the deletion at the target site. SB-E1 and SB-E2 have 24 and 4 bp deletion at the target site, respectively. **(B)** Sanger sequencing of *TalF* EBE edited plants. The plants were named SB-WT (Super Basmati wild type), SB-E3 (Super Basmati edited 3), SB-E4 (Super Basmati edited 4), and SB-E5 (Super Basmati edited 5). SB-E3 and SB-E4 have 18 and 4 bp deletion at the target site, respectively, disrupting *TalF* EBE, whereas SB-E5 had 5 bp deletion at the target site but *TalF* EBE remained intact. **(C)** T7 Endonuclease assay of T_0_ edited and wild type plants. Red arrows showed the bands from edited plants that are absent in wild type plants (E1 = SB-E1, E2 = SB-E2, E3 = SB-E3, E4 = SB-E4, E5 = SB-E5, W1 and W2 = Wild Type Super Basmati). **(D)** Lesions induced by *Xoo* after 14 days post-inoculation on edited and control plants. SB-E1 and SB-E2 has reduced lesion length as compared to control plants. Negative = Plant was inoculated with scissors dipped in water. All the other lines were inoculated with scissors dipped in *Xoo* suspension. **(E)** Average of Percentage disease leaf area (% DLA) of the edited and control plants. The lowest DLA 10.12% was observed in the case of SB-E1 plants where 24 bp deletion was detected. The highest DLA 88.47% was present in IR-24 which was used as a susceptible check.

To determine whether CRISPR-Cas9 was able to cleave and generate DSBs at the target site and repaired it via NHEJ, total genomic DNA was isolated using the CTAB method. Target regions were amplified using PCR and subjected to T7 Endonuclease I (T7EI) mutation detection analysis which degrades single-stranded regions of non-complementarity resulting from indels; these regions are then detected by sequencing. The 457 bp target region from T_0_ was digested with T7E1 and the expected bands (200 and 250 bp approximately) were observed in all five edited lines (Figure 3C). The wild type plants did not show these bands and thus confirmed editing in five lines. Moreover, to further confirm the editing, a 457 bp promoter fragment of the *OsSWEET14* gene from all transgenic plants was sent for Sanger sequencing. We were successful in obtaining two transgenic lines with a mutated promoter fragment for gRNA1 (targeting *AvrXa7* and *PthXo3*). Out of these two lines, one line had 24 bp deletion while the second line showed 4 bp deletion at the target site (Figure 3A). We were unable to obtain transgenic lines for gRNA2. All those lines that were edited with gRNA2 had intact EBE of *TalC*. Out of 51 transgenic lines for gRNA3, we were able to obtain three lines with 4, 5, and 18 bp deletions at the target site. Thus, in two of the three lines (having 4 and 18 bp deletions), we successfully disrupted the EBE of *TalF*. In the third line, 5 bp deletion was not able to disrupt the EBE of *TalF* (Figure 3B). These results showed that CRISPR-Cas9 was able to do editing at the target site while no such activity was observed in the control plants. The nature of mutations was also observed in edited lines. We obtained only one biallelic mutation in SB-E1 while the rest of the mutations were mono-allelic (Supplementary Table S2). However, the edits were transmitted successfully to their progeny.

### Bacterial Blight Resistance Assays

The edited lines were challenged with a prevalent *Xoo* strain via the leaf clip inoculation method. The strain has *AvrXa7* TALE. The presence of *AvrXa7* was not only confirmed by sequencing but the same strain was screened against a rice cultivar (IRBB7) which showed resistance against this strain. IRBB7 has Xa7 gene, which provides resistance against *Xoo* isolates with TALE *AvrXa7* (Yang et al., 2000). This strain was tested to induce the *OsSWEET14* gene. It was able to induce *OsSWEET14*. Therefore, this strain was selected for inoculating edited lines. The wild type Super Basmati rice was used as the control and IR24 (IRRI Line) as a susceptible check. All the inoculated lines were kept at 30°C in a greenhouse with 85% humidity. Two plants from the control and T_0_ edited lines, whereas nine plants from control and T_1_ edited lines were inoculated. The percentage disease leaf area (%DLA) for three inoculated leaves from each plant was calculated (Figure 3E and Supplementary Tables S3, S4). After 14 days post-inoculation, plants were observed for bacterial blight infection. There were significant differences in the rate of infection among edited and control lines. The disease area among edited and control lines were compared in terms of %DLA covered by infection. In *AvrXa7/PthXo3* edited lines, the incidence of infection was very low as compared to the wild type Super Basmati and IR24 lines (Figure 3D). The lowest DLA (10.12%) covered by infection was seen in the case of Super Basmati edited line 1 (SB-E1) in which 24 bp deletion in overlapping EBEs of *AvrXa7* and *PthXo3* occurred. The other variant, SB-E2, showed 14.4% DLA, which was also lower than the control plants. SB-E3 and SB-E4, with disrupted *TalF* EBE and intact *AvrXa7*/*PthXo3* EBE, showed 40 and 41% DLA, respectively, when inoculated with the same strain (possessing only *AvrXa7*). 48 and 88% disease incidence were observed for wild type Super Basmati and IR24, respectively. This confirmed our hypothesis that editing EBEs in the promoter of the *OsSWEET* genes provides resistance against corresponding TALEs.

### Relative *OsSWEET14* Induction by *Xoo* Strain

**FIGURE 4:**
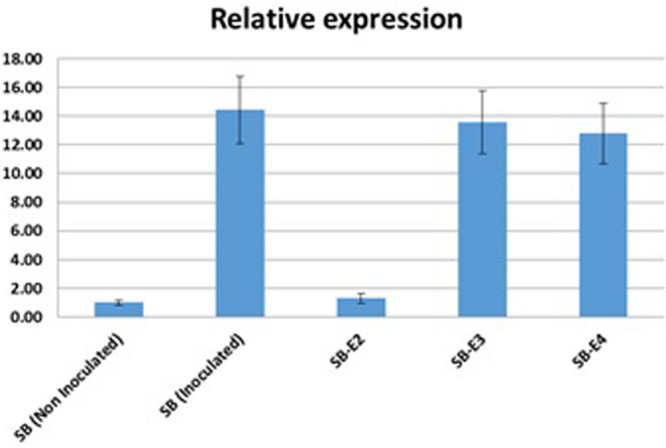
*OsSWEET14* Induction by *Xoo* strain: Relative mRNA levels (2^–ΔΔCt^) of *OsSWEET14* in leaves of wild type and edited rice lines were compared. qRT-PCR was conducted in wild type Super Basmati and edited lines (SB-E2, SB-E3, SB-E4). Samples were harvested after 24 h of Inoculation with a locally virulent *Xoo* strain (mean ± s.e.m., *n* = 3 leaf samples from biological replicates) with expression normalized to rice *SPS* levels; repeated independently three times with comparable results.

*Xoo* strains encode different TALEs to activate endogenous *SWEET* genes for a successful infection. The *OsSWEET14* activation by the *Xoo* strain in wild type Super Basmati Rice and edited lines were determined using real-time quantitative PCR. Expression of *OsSWEET14* was induced by inoculating both wild type and edited lines by the *Xoo* strain. Five lines [(1) Non-inoculated wild type Super Basmati, (2) Inoculated wild type Super Basmati, (3) Inoculated edited line SB-E2, (4) Inoculated SB-E3, and (5) Inoculated SB-E4] were selected to compare *OsSWEET14* activation by *Xoo*. Upon infiltration with the *Xoo* strain, the expression of *OsSWEET14* was considerably high in WT-SB as compared to non-inoculated WT-SB (Figure 4). The edited line (SB-E2) carrying a mutation for *AvrXa7*, showed very low expression of *OsSWEET14.* Whereas in the SB-E3 and SB-E4 lines (carrying edited *TalF* but intact *AvrXa7)*, the strain was still able to induce *OsSWEET14*. This clearly indicates that the *Xoo* strain was able to induce *OsSWEET14* expression to establish successful infection in Super Basmati rice. In addition, the induction of *OsSWEET14* expression in SB-E3 and SB-E4 edited lines clearly showed that the *Xoo* strain contained *AvrXa7* TALE to infect Super Basmati rice. As SB-E2 edited line has mutated EBE for *AvrXa7* and the strain was unable to induce *OsSWEET14* expression. As a result, relative expression of *OsSWEET14* was significantly lower in the SB-E2 edited line as compared to other inoculated lines.

### Phenotyping

**FIGURE 5:**
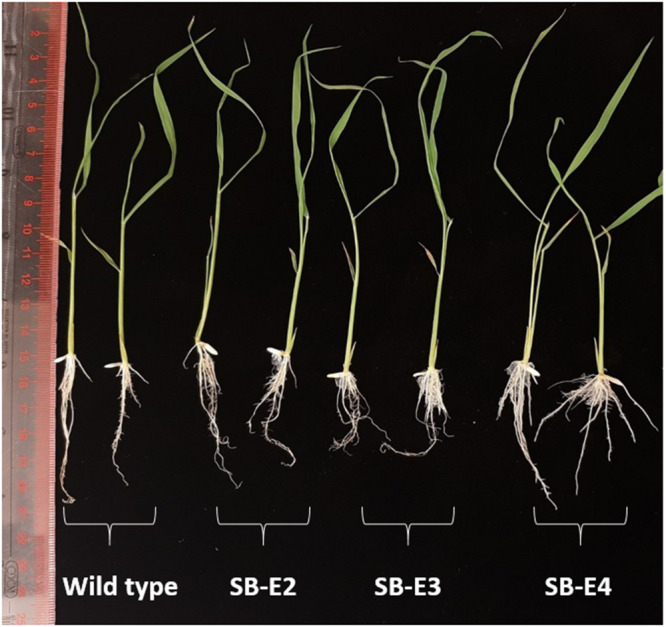
Root length and shoot length of wild type and edited plants. Edited plants show normal root and shoot length just like wild type plants (two representative plants are shown in figure out of three).

The plants were carefully observed for any phenotypic changes. All the edited plants showed a normal phenotype and were fertile just like the wild type plants. The harvested seeds also showed a normal phenotype. The germination was checked for seeds from all the edited lines (Except SB-E1) and seeds showed normal germination (Supplementary Figure S5). The shoot and root lengths were also normal for the edited lines (Figure 5).

## Discussion

In a global scenario, bacterial blight is a destructive disease of rice, and in Asia specifically, it severely damages crop yield. Traditionally, various approaches have been used to develop resistance in rice cultivars which include the introgression of resistance genes (R genes), host-derived resistance, and loss of susceptibility through mutation in recessive R genes (Dou and Zhou, 2012; Kim et al., 2015; Busungu et al., 2016; Li et al., 2019). Despite the effectiveness of R genes, the constant introduction of R-genes in rice breeding programs may result in the emergence of new *Xoo* strains, which can overcome R gene-mediated resistance (Ji et al., 2016). With the advent of new technologies like genome editing, various strategies have been employed, e.g., TALENs and CRISPR-Cas9 to develop disease resistance (Khan et al., 2014). Here in this study we used CRISPR-Cas9 technology to develop disease resistance in Basmati rice.

One approach which can be used to remove disease predisposition is to mutate the susceptible portion of the genome (Iyer-Pascuzzi and McCouch, 2007; Hutin et al., 2015a; Kourelis and van der Hoorn, 2018; Zaidi et al., 2018). The idea to mutate the bacterial blight susceptible region of the rice genome sparked from the presence of three naturally occurring recessive R genes, i.e., *xa13*, x*a25*, and *xa41 (t)*. These are mutated alleles of *OsSWEET11*, *OsSWEET13*, and *OsSWEET14*, respectively, which offers resistance against their respective TALEs present in the *Xoo* strain (Chu et al., 2006; Yang et al., 2006; Yuan et al., 2009; Hutin et al., 2015b). It has been reported that *Xa41*(t) (having 18 bp deletion) offers broad-spectrum resistance against a large collection of *Xoo* strains (Hutin et al., 2015b). Recently it was reported that some *Xoo* strains still cause infection in rice despite R genes (Carpenter et al., 2018; Doucouré et al., 2018). These failures have prompted plant genome engineers to develop resistance against this rapidly evolving pathogen by mutating susceptibility genes. TALENs have been employed in the past, but extensive protein engineering makes developing broad-spectrum immunity against bacterial blight difficult (Li et al., 2012). The prokaryotic immune system (CRISPR-Cas9) was customized and used for editing susceptibility genes in rice (Jiang et al., 2013). In the current study this customized tool was used for editing the *OsSWEET14* gene’s promoter of Basmati, to obtain resistance against bacterial blight.

Basmati rice is famous for its long grain and aroma and none of the natural mutations are reported in *OsSWEET14* for Super Basmati rice. For the *OsSWEET13* gene, one deletion and substitutions are naturally present in the promoter which disrupts all the EBE variants of *PthXo2* TALE, i.e., *PthXo2A*, *PthXo2B*, and *PthXo2C* (Zaka et al., 2018; Oliva et al., 2019). It was reported that in the majority of cases, *Xoo* strains induce the expression of *OsSWETT14* for the onset of infection because it has the EBEs of four different TALEs. *OsSWEET14* was targeted previously as a single target as well as in combination with other *OsSWEET* genes to create broad-spectrum resistance in different rice cultivars (Blanvillain-Baufumé et al., 2017; Oliva et al., 2019; Xu et al., 2019). They have developed different lines with edited EBEs of the *OsSWEET14* gene’s promoter. Each EBE edited line provided resistance against specific TALE. In the present study, the *OsSWEET14* gene’s promoter was selected to modify the respective EBEs of four TALEs via CRISPR-Cas9. We have successfully mutated EBEs of three TALEs (*AvrXa7, PthXo3*, and *TalF*). The edited rice lines (SB-E1, SB-E2, SB-E3, and SB-E4) have modified alleles of *OsSWEET14*. Among these four edited lines, SB-E1 and SB-E2 did not respond to *AvrXa7/PthXo3* to establish a successful infection. The new germplasm created in this way showed resistance against a locally virulent *Xoo* strain. These results indicated that CRISPR-Cas9 technology can also be employed on Basmati rice to create resistance against bacterial blight.

Blanvillain-Baufumé et al. (2017) created the allele library of *OsSWEET14* for developing resistance against bacterial blight. The *OsSWEET14* gene’s promoter was targeted using TALENS, and they observed 51% editing efficiency. In the current study the EBEs of the *OsSWEET14* gene’s promoter were targeted using CRISPR-Cas9 and we were able to obtain only 9% editing efficiency. All the editing events were in close proximity to the predicted cleavage site by Cas9 which is three base pairs upstream of the PAM sequence. This indicates the specificity of this editing system. However, we observed low editing efficiency as compared to the previous report of editing the *OsSWEET14* gene’s promoter. The reason for low editing efficiency could be the difference in the editing tool (TALENs) and rice variety as they have used a non-Basmati background. The editing efficiency can be improved by using Cas9 fusion with chromatin-modulating peptides (CMPs), derived from high mobility group proteins. This fusion exhibited many folds improved activity (Ding et al., 2019). Thus, such type of improved systems can be tested in elite varieties, like Super Basmati, to check improvement in editing efficiency. Blanvillain-Baufumé et al. (2017) obtained mixed events (insertion/deletion) at the target sites including the insertion of 22 bp and deletions of up-to 51 bp, whereas another study by Jiang et al. (2013) reported only deletions at the target site. In our case, we were only able to obtain deletions at the target sites, because the repairing of DSB (introduced by CRISPR-Cas9) was done through a random NHEJ process. Therefore, obtaining deletions, insertions, or point mutations at the target site is solely dependent on the host repair machinery.

The diversity of TALEs present in different *Xoo* strains was studied previously by analyzing *Xoo* genome sequence data. Most of the Asian strains had *AvrXa7* (*OsSWEET14*) and *PthXo2* (*SWEET13*) (Oliva et al., 2019). Sequencing data of the most prevalent *Xoo* strain infecting Basmati showed that this strain had *AvrXa7* for infecting Basmati. The induction of *OsSWEET14* was also checked by the tested *Xoo* strain and RT-qPCR results confirmed *OsSWEET14* induction by the tested *Xoo* strain. So, targeting the promoter of *OsSWEET14* in Super Basmati is very effective in generating broad-spectrum resistance. However, this strategy can be modified depending on the TALEs present in *Xoo* strains and their respective EBEs in rice cultivars. More work is still required, however, in order to deal with bacterial blight by characterizing maximum *Xoo* isolates. Some *Xoo* TALEs also target *OsSWEET13* and *OsSWEET11* for the onset of infection. But the isolates which we have characterized were mostly targeting *OsSWEET14*, which agrees with studies reported by Blanvillain-Baufumé et al. (2017).

In a previous report it was shown that Asian *Xoo* strains did not have *TalF* (Oliva et al., 2019). Our study also supports their finding because our locally isolated strain did also not possess *TalF*. The selected *Xoo* strain was inoculated on all the edited lines and disease development was recorded. The symptoms were reduced on *AvrXa7* edited lines. When *TalF*-edited lines were challenged with the same *Xoo* strain, the symptoms were not reduced. This was further confirmed by RT-qPCR of the control and edited lines. The relative expression of *OsSWEET14* was similar in wild type SB and *TalF* edited lines (SB-E3, SB-E4), whereas the strain was unable to induce *OsSWEET14* in the *AvrXa7* edited line (SB-E2). This showed that the strain has *AvrXa7* to infect Super Basmati. These findings confirmed our hypothesis that targeting EBEs in the promoter region only provides specific resistance to their corresponding TALE present in the *Xoo* strain.

Mutations in susceptibility genes can also have side effects on normal plant physiology (van Schie and Takken, 2014). In a previous study, rice plants with a *OsSWEET14* TDNA insertion mutant had smaller seeds as compared to wild type plants, although it showed resistance against *Xoo* strains (Antony et al., 2010). In contrast, *OsSWEET14* EBE edited rice plants were no longer susceptible to *Xoo* and showed normal growth (Li et al., 2012). In the current study the edited lines were visually observed during the growth period and after harvest. All the edited lines showed no detectable growth defects in greenhouse conditions except for SB-E1. In SB-E1 seed filling was disturbed. The reason could be attributed to a large deletion in the promoter region. This also indicates the role of *OsSWEET* genes in seed filling (Sosso et al., 2015; Yang et al., 2018; Hu et al., 2019). However, further investigation is required in this regard. Thus, in comparison to former studies, we came across both types of results, and out of four EBE mutated lines we observed abnormality in only one edited line.

There are some previous reports of CRISPR-Cas9 mediated genome editing of rice to develop resistance against bacterial blight. Editing in the promoter fragment of *OsSWEET* genes was reported using CRISPR-Cas9 to develop resistance against bacterial blight (Oliva et al., 2019; Xu et al., 2019). But the majority of previous genome editing was performed in rice cultivar kitake (japonica) and cannot be used for breeding programs of Basmati rice. Recently, genome editing via CRISPR-Cas9 was performed to mutate EBEs of *OsSWEET11*, *OsSWEET13*, and *OsSWEET14* to create resistance in Indica cultivar (IR-64 and Ciherang-Sub1). These cultivars can be used by breeders of Asia and Africa in breeding programs but there was no report on creating resistance in the Basmati cultivars. Therefore, we performed genome editing in the elite Super Basmati rice cultivar to be incorporated into breeding programs. The present study was designed to establish CRISPR-Cas9 mediated genome editing in Basmati rice. To the best of our knowledge, this is the first report of genome editing in Basmati rice using the CRISPR-Cas9 toolbox.

This study shows the potential of CRISPR-Cas9 based genome editing in elite Super Basmati rice. However, there is still more work needed to deal with bacterial blight, by characterizing the maximum of local *Xoo* isolates, because some *Xoo* TALEs also target *OsSWEET13* and *OsSWEET11*. So, to create broad-spectrum resistance against all the native *Xoo* strains, multiplexing can also be done in Basmati. Further, a study can be planned to establish multiplex genome editing against bacterial blight by simultaneously targeting multiple EBEs in the promoter regions of *OsSWEET* genes, which will ultimately result in reducing bacterial blight. This approach of dealing with bacterial blight by targeting the promoter of *OsSWEET* genes will not prevent the adaptation of pathogens. The strength of this method also depends on the ability of local *Xoo* strains to acclimate to recessive resistance alleles. To develop durable resistance against pathogens, it is better to create major changes in EBEs of the *OsSWEET* gene’s promoter. Combining these edited alleles with locally effective resistance genes can be a more effective way to reduce disease pressure. In conclusion, by understanding TALE interaction with EBEs of *OsSWEET14* genes, we were able to create resistance against a corresponding *Xoo* strain.

Genome editing can have off-target effects. The gRNAs used in this study were analyzed for off-targeting against the rice genome available in the database. All the gRNAs did not have any off-targets. Furthermore, if any off-target mutations are still present as a result of genome editing or tissue culturing, they will be eliminated during crossing. Full genome sequencing will be required to confirm any off-targeting. Finally, our initial data shows the potential of CRISPR-Cas9 based genome editing in elite Basmati cultivar. However, the establishment of a transgene-free genome editing protocol for targeting multiple EBEs simultaneously is still needed in Super Basmati rice to create broad-spectrum resistance against a large collection of *Xoo* strains. Such transgene-free genome-edited elite lines created in this way can be added in breeding programs.

## Conclusion

Our results show that targeting EBEs of respective TALEs, employing the CRISPR-Cas9 approach, can provide highly selective and promising immunity against bacterial blight.

## Data Availability Statement

All datasets generated for this study are included in the article/Supplementary Material.

## Author Contributions

SM planned the work. KZ, MK, and IA planned the experiments. KZ performed all the experiments and wrote the manuscript. MK and IA edited and finalized the manuscript. ZM helped establishing tissue culture of Super Basmati. SY and KE performed the resistance assays against *Xoo*. MA provided the seeds for this study and give critical suggestions.

## Conflict of Interest

The authors declare that the research was conducted in the absence of any commercial or financial relationships that could be construed as a potential conflict of interest.

## Footnotes

1 http://www.rgenome.net/cas-offinder/
