# Supplementary material for: Precise CRISPR-Cas9 Mediated Genome Editing in Super Basmati Rice for Resistance Against Bacterial Blight by Targeting the Major Susceptibility Gene
Source: Front Plant Sci. 2020 Jun 12;11:575. doi: 10.3389/fpls.2020.00575 (PMC7304078; doi:10.3389/fpls.2020.00575)
Supplement: Supplementary file 1 [file Data_Sheet_1.pdf]

Supplementary Figures and Table

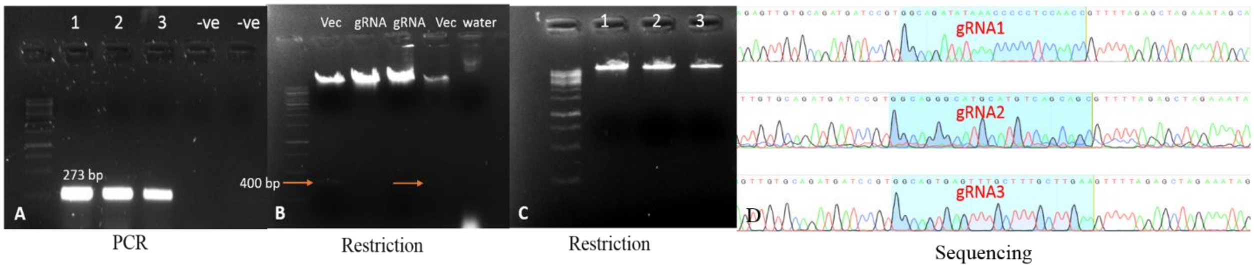

Figure S1| A-PCR confirmation of constructs followed by restriction (B and C). D- Confirmation of constructs by Sanger sequencing

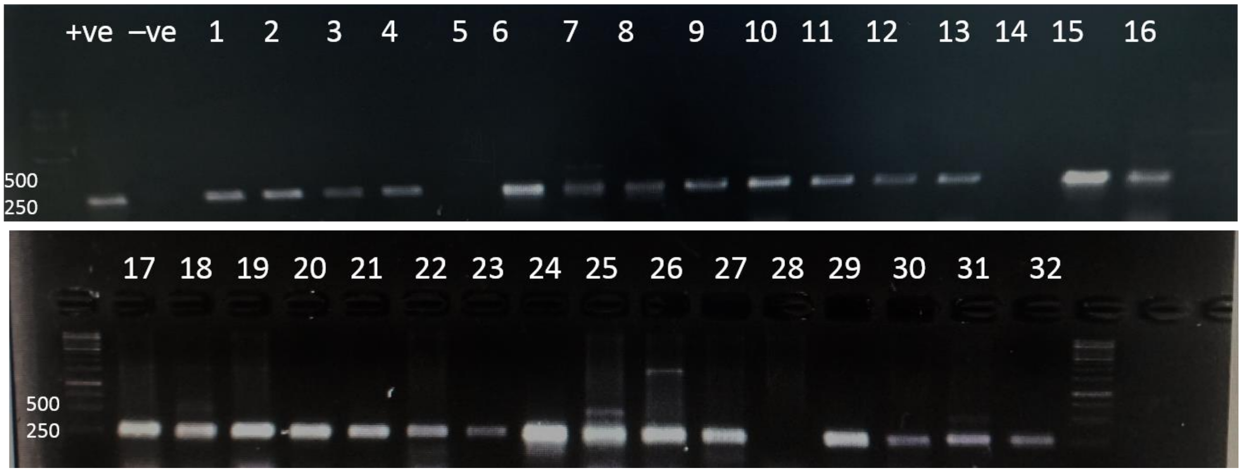

Figure S2| PCR confirmation of plants. The band size of 273bp showed the presence of constructs in the plants

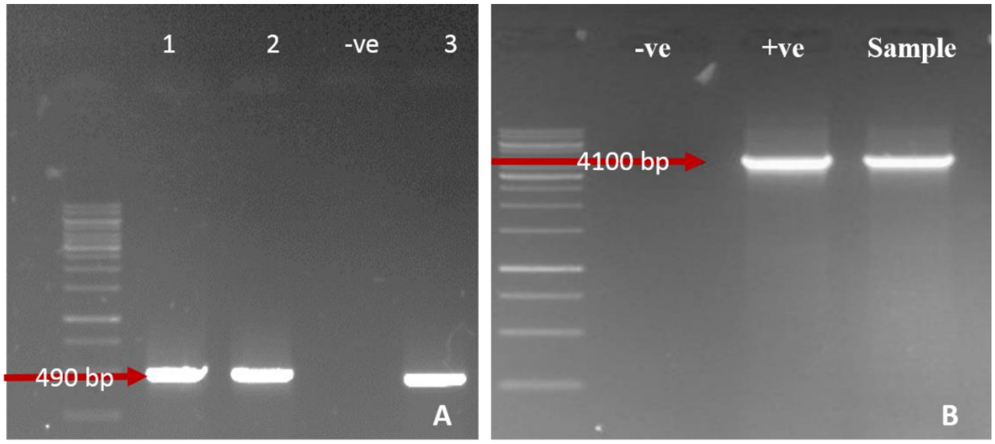

Figure S3| Cas9 Confirmation: **(A)** The presence of Cas9 was confirmed in all the three constructs. Initially with Cas9-F AGCATCGGCCTGGACATCGGC and Cas9 R-GGAAGTGGCCCCGGAACTTG primers (490bp amplification)

1= Construct having gRNA1

2= Construct having gRNA2

3= Construct having gRNA3

-ve= PCR without construct

(B) Full length Cas9 was amplified from construct and transgenic plant sample using Cas9-full F-GACAAGAAGTACAGCATCGG and Cas9-full R- CGCCTCCCAGCTGAGACAGG primers (4100bp Amplification). The full length Cas9 was present in plant samples.

-ve= PCR without construct or plant sample

+ve= PCR with construct having Cas9

Sample= PCR from Plant sample of edited line

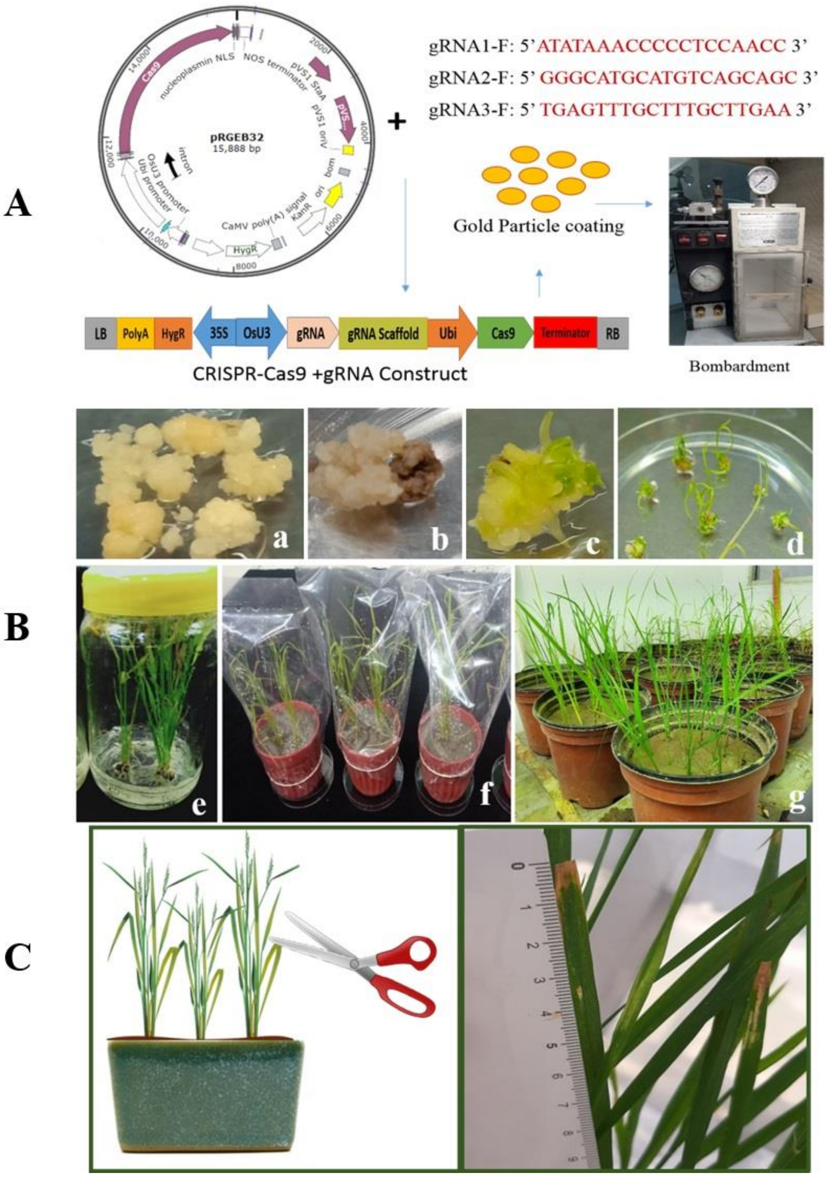

Figure S4 | (A) Construct development and transformation (**B**) Transgenic plant development- (a) Embryogenic calli selected for transformation (b) Selection of transformed calli on selection media (c) Regenerated callus on regeneration media (d) Shoots development (e) Roots formation on rooting media (f) Plants shifted in smaller pots (g) Established plants in bigger pots in soil (**C**) Leaf clipping with *Xoo* strain and lesion measurement to screen plants against bacterial blight.

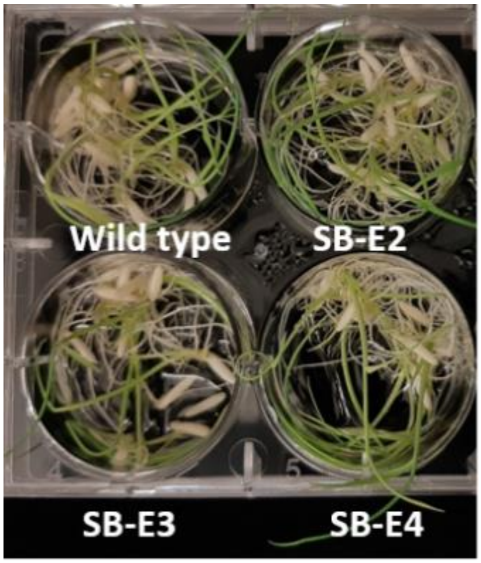

Figure S5| Germination of wild type and edited plants. Ten to twelve seeds were places in sterilized water and their germination was recorded. The germination of the seeds was normal.

Table S1| Primers used in Study

Sr. No.	Purpose	Primer set	Product Length (bp)
1	To amplify promoter region of *SWEET14*	*Os*P-F: ATTGGCACTTTCTGTCATGCATG<br>*Os*P-R: GCAAGATCTTGATTAACTAGCTAGC	457
2	Partial Cas9	Cas9-F: AGCATCGGCCTGGACATCGGC<br>Cas9 R: GGAAGTGGCCCCGGAACTTG	490
3	Full Cas9	Cas9-full F: GACAAGAAGTACAGCATCGG<br>Cas9-full R: CGCCTCCCAGCTGAGACAGG	4100
4	For RT-qPCR of *OsSWEET14*	*SWEET14*- RT-F TGGCATGTCTCTTCAGCATC<br>*SWEET14*- RT-R CGCGTAGTAGATCCACAGCA	187
5	Internal control for RT-qPCR	SPS- F: GCCATGGATTACATATGGCAAGA<br>SPS- R: ATCTGTTTACTCGTCAAGTGTCATCTC	287

Table S2| Editing efficiency of CRISPR-Cas9 in basmati rice using biolistic transformation

gRNA used	Exp. No.	No. of calli bombarded	No. of calli survived on selection	No. of plants produced	No. of plants Recovered	No. of edited plants	*Bi-allelic/ Mono-allelic editing	**Editing efficiency (%)
gRNA1	1	1500	102	30	25	1	B	4
	2	1000	75	15	9	0	N	0
	3	1000	95	12	11	1	M	9.1
gRNA2	1	1500	88	19	15	0	N	0
	2	1000	72	11	10	0	N	0
	3	1000	65	9	7	0	N	0
gRNA3	1	1500	92	22	18	1	M	5.6
	2	1200	101	32	27	2	M	7.4
	3	1000	62	18	12	0	N	0
Total		10700	752	168	134	5	1B & 4M	3.7

** (No. of edited plants/No. of recovered plants)*100

*B= Biallelic, M= Monoallelic, N= No editing

Table S3| Screening against BLB (T_0 generation)

Type of editing	Plant	Leaf #	Percentage Disease Leaf Area(DLA) (cm)	%DLA Mean ±S.E.M	%DLA of 2 plants Mean ±S.E.M
Wild type	Negative control-1	I	1.6	1.1±0.2	1.1±0.04
		II	0.9		
		III	0.8		
	Negative control-2	I	0.9	1.2±0.7	
		II	1.5		
		III	1.3		
Disruption at EBE of A*vrXa7* and *PthXo3*	SB-E1 (1)	I	8.3	9.7±0.7	10.1±0.32
		II	10		
		III	11		
	SB-E1 (2)	I	9.2	10.5±0.6	
		II	10.8		
		III	11.4		
	SB-E2 (1)	I	14	14.4±0.2	14.6±0.16
		II	13.8		
		III	14.5		
	SB-E2 (2)	I	15.3	14.8±0.2	
		II	14.6		
		III	14.6		
Wild Type	SB-W1 (1)	I	50	46.6±1.6	48.1±1.22
		II	45		
		III	45		
	SB-W1 (2)	I	50	49.6±2.6	
		II	54		
		III	45		
Disruption at EBE of *TalF*	SB-E3 (1)	I	44	40±2.6	40±0.04
		II	41		

		III	35		
	SB-E3 (2)	I	37.5	40.1±1.9	
		II	44		
		III	38.8		
	SB-E4 (1)	I	45	41±2.08	41±0.21
		II	40		
		III	38		
	SB-E4 (2)	I	40	41.5±4.2	
		II	50		
		III	34.5		
Wild Type (susceptible control)	IR24 (1)	I	80	88.5±4.3	88±0.48
		II	91.6		
		III	94		
	IR24 (2)	I	90.9	87.3±1.8	
		II	84.6		
		III	86.6		

Negative Control: Super Basmati plant inoculated with scissors dipped in distilled water to check injury induced by scissors.

SB-E1 and SB-E2: Have edited *AvrXa7* {SB-E1 (24 bp deletion), SB-E2 (4 bp deletion)}

SB-W1: Non edited wild type Super Basmati rice

SB-E3 and E4: Have edited *TalF* but intact *AvrXa7* {SB-E3 (18 bp deletion), SB-E4 (4 bp deletion)}

IR-24: Susceptible to almost all the *Xoo* strains and was used as susceptible control

S.E.M= Standard error of Mean

Table S4| Screening against BLB (T_1 generation)

**1. Wild Type Super Basmati**			
**Batch #**	**Plant #**	**Mean DLA of 3 leaves (%)**	**DLA of 3 plants (%)** (Mean ± S.E.M)
Batch-1	1	40	45±2.8
	2	50	
	3	45	
Batch-2	1	58	53±3.6
	2	46	
	3	55	
Batch-3	1	60	55±2.8
	2	50	
	3	55	
**2. SB-E2**			
**Batch #**	**Plant #**	**Mean DLA of 3 leaves (%)**	**DLA of 3 plants (%)** (Mean ± S.E.M)
Batch-1	1	15	16.3±1.8
	2	20	
	3	14	
Batch-2	1	22	17.6±2.3
	2	17	
	3	14	
Batch-3	1	21	16.6±2.1
	2	14	
	3	15	
**3. SB-E3**			
**Batch #**	**Plant #**	**Mean DLA of 3 leaves (%)**	**DLA of 3 plants (%)** (Mean ± S.E.M)
Batch-1	1	39	41.6±1.7

	2	41	
	3	45	
Batch-2	1	42	41.3±1.7
	2	44	
	3	38	
Batch-3	1	39	40.3±0.8
	2	42	
	3	40	
**4. SB-E4**			
**Batch #**	**Plant #**	**Mean DLA of 3 leaves (%)**	**DLA of 3 plants (%)** (Mean ± S.E.M)
Batch-1	1	46	45±1.6
	2	49	
	3	40	
Batch-2	1	39	44±3.1
	2	45	
	3	50	
Batch-3	1	39	45±3.7
	2	45	
	3	52	
**5. IR24**			
**Batch #**	**Plant #**	**Mean DLA of 3 leaves (%)**	**DLA of 3 plants (%)** (Mean ± S.E.M)
Batch-1	1	88	87±2.9
	2	92	
	3	82	
Batch-2	1	90	89±2.1
	2	92	
	3	85	
Batch-3	1	78	85±3.7

	2	86	
	3	91	
**6. Negative Control**			
**Batch #**	**Plant #**	**Mean DLA of 3 leaves (%)**	**DLA of 3 plants (%)** (Mean ± S.E.M)
Batch-1	1	1.5	1.5±0.2
	2	2	
	3	1.1	
Batch-2	1	2.1	1.9±0.4
	2	2.5	
	3	1	
Batch-3	1	1	1.2±0.3
	2	0.8	
	3	2	

SB-W1: Non edited wild type Super Basmati rice

SB-E2: Have edited *AvrXa7*

SB-E3 Have edited *TalF* but intact *AvrXa7*

SB-E4: Have edited *TalF* but intact *AvrXa7*

IR-24: Susceptible to almost all the *Xoo* strains and was used as susceptible control

Negative Control: Super Basmati plant inoculated with scissors dipped in distilled water to check injury induced by scissors.

S.E.M= Standard error of mean
